# Supplementary material for: Auto-inhibitory intramolecular S5/S6 interaction in the TRPV6 channel regulates breast cancer cell migration and invasion
Source: Commun Biol. 2021 Aug 19;4:990. doi: 10.1038/s42003-021-02521-3 (PMC8376870; doi:10.1038/s42003-021-02521-3)
Supplement: Supplementary file 2 — Supplementary Information [file 42003_2021_2521_MOESM2_ESM.pdf]

Supplementary Information

Auto-inhibitory intramolecular S5/S6 interaction in the TRPV6 channel regulates breast cancer cell migration and invasion

Cai et al.

Fig. S1

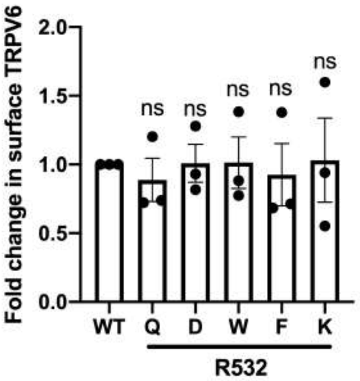

Supplementary Figure 1. Bar charts showing the surface expression of TRPV6 mutants in comparison with WT TRPV6. Shown values were averages from three independent experiments. ns, not significant.

Fig. S2

Accession	Name	S5	S6 / TRP
Q9H1D0	TRPV6_HUMAN	KMIFGDLM**R**FCWLMAVVILGFASAFYIIFQ	AFAIIATLLMLNLLIAMMG**D**THWRVAHERDELWRAQIVATTVMLERKLPR
Q9NQA5	TRPV5_HUMAN	KMIFGDLM**R**FCWLMAVVILGFASAFYIIFQ	AFAIIATLLMLNLFIAMMG**D**THWRVAQERDELWRAQVVATTVMLERKLPR
Q8NER1	TRPV1_HUMAN	KMILRDLC**R**FMFVYIVFLFGFSTAVVTLIE	AYVILTYILLLNMLIALMG**E**TVNKIAQESKNIWKLQRAITILDTEKSFLK
Q9Y5S1	TRPV2_HUMAN	KVILRDLL**R**FLLIYLVFLFGFAVALVSLSQ	AYVLLTYILLLNMLIALMS**E**TVNSVATDSWSIWKLQKAISVLEMENGYWW
Q8NET8	TRPV3_HUMAN	KVILHDVL**K**FLFVYIVFLLGFGVALASLIE	TYVILTFVLLLNMLIALMG**E**TVENVSKESERIWRLQRARTILEFEKMLPE
Q9HBA0	TRPV4_HUMAN	KILFKDLF**R**FLLVYLLFMIGYASALVSLLN	TYIILTFVLLLNMLIALMG**E**TVGQVSKESKHIWKLQWATTILDIERSFPV
Q9H1D0	TRPV6_HUMAN	KMIFGDLM**R**FCWLMAVVILGFASAFYIIFQ	AFAIIATLLMLNLLIAMMG**D**THWRVAHERDELWRAQIVATTVMLERKLPR
Q91WD2	TRPV6_MOUSE	KMIFGDLM**R**FCWLMAVVILGFASAFYIIFQ	AFAIIATLLMLNLLIAMMG**D**THWRVAHERDELWRAQVVATTVMLERKLPR
Q9R186	TRPV6_RAT	KMIFGDLM**R**FCWLMAVVILGFASAFYIIFQ	AFAIIATLLMLNLLIAMMG**D**THWRVAHERDELWRAQVVATTVMLERKLPR
E7EEJ8	TRPV6_DANRE	KSIFGDIT**K**FMWLSIIFLIGSSAALWIFYM	CFSLISNVLLFNLLVAMMS**D**TQWRVTQERDELWRTQVVATTLMLERKLPQ
F6Y562	TRPV6_XENTR	QIIFGDVL**R**FFWLMMVVILGFGTALFVVFQ	PFTLIANLMMMNMLIGMMG**D**THWRVAQERDELWRAQLAAITIMLEGKFPK

Accession	Name	S5	S6
Q7Z2W7	TRPM8_HUMAN	IMLQ**R**MLIDVFFFLFLFAVWMVAF	IYMLSTNILLVNLLVAM**F**GYTVGT
Q7Z4N2	TRPM1_HUMAN	MMIG**K**MMIDMLYFVVIMLVVLMSF	CYLLVANILLVNLLIAV**F**NNTFFE
O94759	TRPM2_HUMAN	IIVK**R**MMKDVFFFLFLLAVWVVSF	LYLLFTNILLLNLLIAM**F**NYTFQQ
Q9HCF6	TRPM3_HUMAN	MMIG**K**MMIDMMYFVIIMLVVLMSF	CYLLVANILLVNLLIAV**F**NNTFFE
Q8TD43	TRPM4_HUMAN	VIVS**K**MMKDVFFFLFFLGVWLVAY	IFLLVANILLVNLLIAM**F**SYTFGK
Q9NZQ8	TRPM5_HUMAN	IVVE**R**MMKDVFFFLFFLSVWLVAY	TFLLVTNVLLMNLLIAM**F**SYTFQV
Q9BX84	TRPM6_HUMAN	TMIA**K**MTANMFYIVIIMAIVLLSF	VYLFVQYIIMVNLLIAF**F**NNVYLD
Q96QT4	TRPM7_HUMAN	MMIG**K**MVANMFYIVVIMALVLLSF	VYLFVQYIIMVNLLIAF**F**NNVYLQ
P48995	TRPC1_HUMAN	QDFG**K**FLGMFLLVLFSFTIGLTQ	VVVIVLTKLLVAMLHKS**F**QLIANH
Q9R244	TRPC2_MOUSE	DDMI**R**FMFILMIILTAFLCGLNN	VMVIVLLNMLIAMITNS**F**QKIEDD
Q13507	TRPC3_HUMAN	KDIF**K**FMVLFIMVFFAFMIGMFI	TMVVVLLNMLIAMINSS**Y**QEIEDD
Q9UBN4	TRPC4_HUMAN	LDIL**K**FLFIYCLVLLAFANGLNQ	ISLVVLLNMLIAMMNNS**Y**QLIADH
Q9UL62	TRPC5_HUMAN	LDIL**K**FLFIYCLVLLAFANGLNQ	ISLVVLLNMLIAMMNNS**Y**QLIADH
Q9Y210	TRPC6_HUMAN	KDIF**K**FMVIFIMVFVAFMIGMFN	TMVIVLLNMLIAMINSS**F**QEIEDD
Q9HCX4	TRPC7_HUMAN	KDIF**K**FMVIFIMVFVAFMIGMFN	TMVVVLLNMLIAMINNS**Y**QEIEED
Q9GZU1	TRPML1_HUMAN	PSVM**R**FCCCVAVIYLGYCFCGWI	FIYMVLSLFIALITGAY**D**TIKHPG
Q8IZK6	TRPML2_HUMAN	PKVL**R**FCACAGMIYLGYTFCGWI	FIYMILSLFIALITDSY**D**TIKKFQ
Q8TDD5	TRPML3_HUMAN	PNVI**R**FCCCAAMIYLGYCFCGWI	FIYMILSLFIALITDTY**E**TIKQYQ
O75762	TRPA1_HUMAN	KTLL**R**STVVFIFLLLAFGLSFY	IFVPIVLMNLLIGLAVG**D**IAEVQK
Q8BLA8	TRPA1_MOUSE	KTLL**R**STGVFIFLLLAFGLSFY	MFVPIVLMNLLIGLAVG**D**IAEVQK
Q6RI86	TRPA1_RAT	KTLL**R**STGVFIFLLLAFGLSFY	MFVPIVLMNLLIGLAVG**D**IAEVQK
Q7Z020	TRPA1_DROME	QTLI**K**VLMVFSILIIAFGLAFY	ILMPILLMNLLIGLAVG**D**IESVRR
F1Q2M0	TRPA1_CANLF	KTLL**R**STVVFVFLLLAFGLSFY	MFVPIVLMNLLIGLAVG**D**IAEVQK
I3LEM4	TRPA1_PIG	KTLL**K**STVVFVFLLVAFGLSFY	MFVPIVLMNLLIGLAVG**D**IAEVQK
G5E522	TRPA1_BOVIN	KTLL**K**STIVFVFLLVAFGLCFY	MFVPIVLMNLLIGLAVG**D**IAEVQK
Q18297	TRPA1_CAEEL	KTFF**R**FFPVFVLFIIAFSSSFY	IIMTILLMNLLVGLAVD**D**IKGVQE
Q13563	PKD2_HUMAN	SRCA**K**DLFGFAIMFFIIFLAYA	VFFMFFILLNMFLAIIN**D**TYSEVK
Q9P0L9	PK2L1_HUMAN	ARCA**K**DILGFAVMFFIVFFAYA	VFFVFFVLLNMFLAIIN**D**TYSEVK
Q9NZM6	PK2L2_HUMAN	SRCV**K**DIVGFAIMFFIIFFAYA	IFFVFFVLLNMFLAIIN**D**TYSEVK

Supplementary Figure 2. Sequence alignments of helices S5 and S6 in TRP channels. Uniprot accession numbers are included. Residues corresponding to R532 and D620 in human TRPV6 are in bold and underlined.

Fig. S3

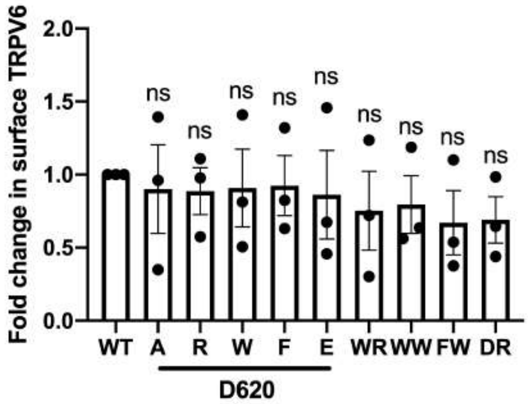

Supplementary Figure 3. Bar charts showing the surface expression of TRPV6 mutants in comparison with WT TRPV6. Shown values were averages from three independent experiments. WR: R532W/D620R; WW: R532W/D620W; FW: R532F/D620W; DR: R532D/D620R. ns, not significant.

Fig. S4

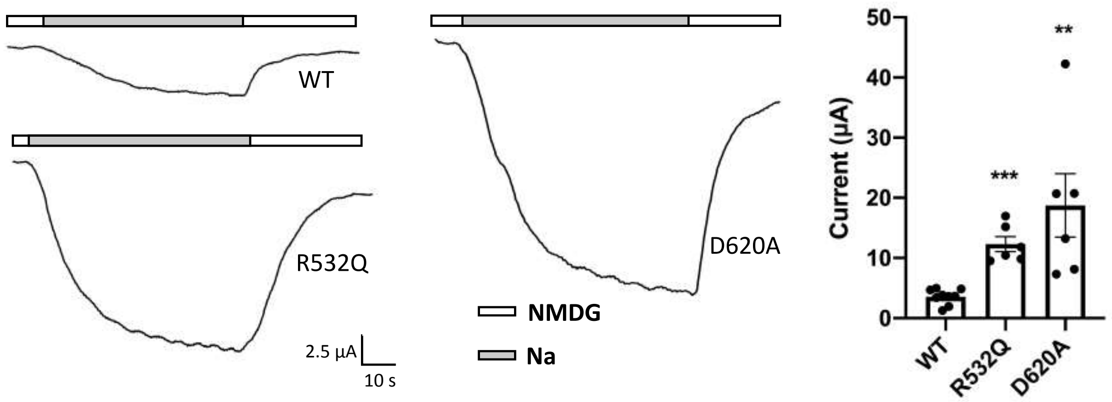

Supplementary Figure 4. Na currents mediated by WT and mutant TRPV6. Left panel: bar charts showing Na currents from oocytes expressing WT or an indicated mutant clamped at -50 mV. Shown values were averages from N=6-9 oocytes. Right panel: representative current traces at -50 mV from oocytes expressing WT or an indicated mutant. **, $p < 0.01$; ***, $p < 0.001$. Experiments were independently performed using three batches of oocytes.

Fig. S5

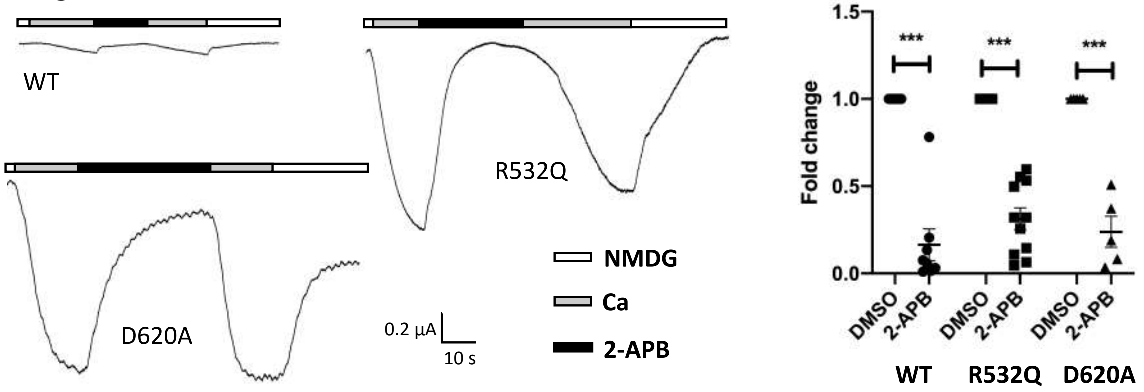

Supplementary Figure 5. Na currents mediated by WT and mutant TRPV6. Left panel: representative traces from oocytes expressing WT or indicated mutants. Right panel: data showing analysis summarizing 2-APB inhibition on WT or indicated mutants. Shown values were averages from N=5-11 oocytes. ***, $p < 0.001$.

Fig. S6

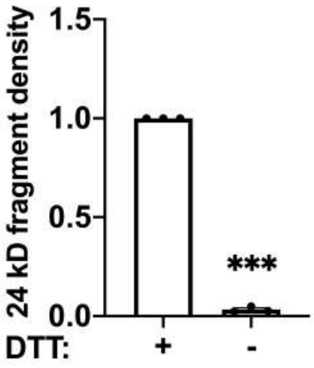

Supplementary Figure 6. Bar charts showing the density of 24 kD fragment using TRPV6 R532C/D620C$_{TEV}$ with and without DTT treatment. Shown values were averages from three independent experiments. ***, $p < 0.001$.

Fig. S7

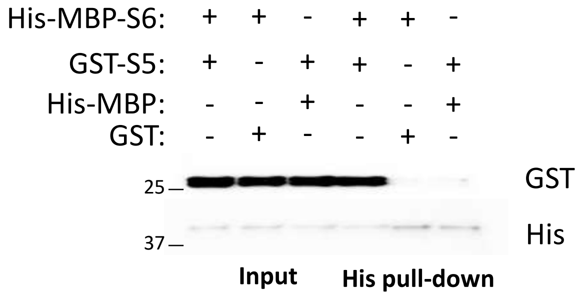

Supplementary Figure 7. Interactions between S5 helix with MBP and between S6 helix with GST, respectively. Representative *in vitro* binding data obtained using His pull down showing interaction between two indicated fragments (by ‘+’).

Fig. S8

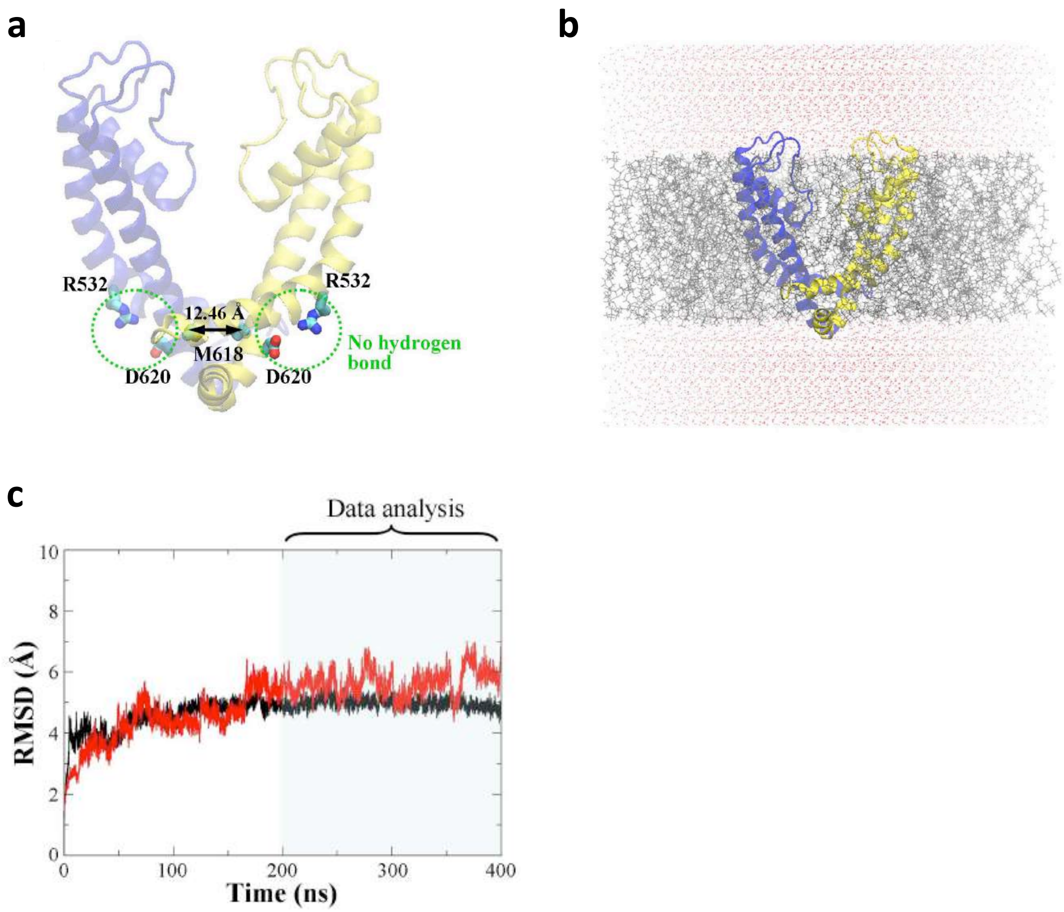

Supplementary Figure 8. Initial simulation model and root mean square deviation (RMSD) for the Cα atoms of TRPV6 as a function of time. (a) The pore region of TRPV6 (G511~T640) is embedded in POPC lipid bilayer. For clarity, only two diagonal monomers are shown. In the initial model, the distance between the two diagonal M618 is 12.46 Å and there is no hydrogen bond between R532 and D620. (b) TRPV6 pore region shown by two monomers embedded in POPC lipid layer. (c) RMSD results show that the simulation reaches equilibrium after 200 ns. Thus, the last 200 ns trajectories were used for data analysis.

Fig. S9

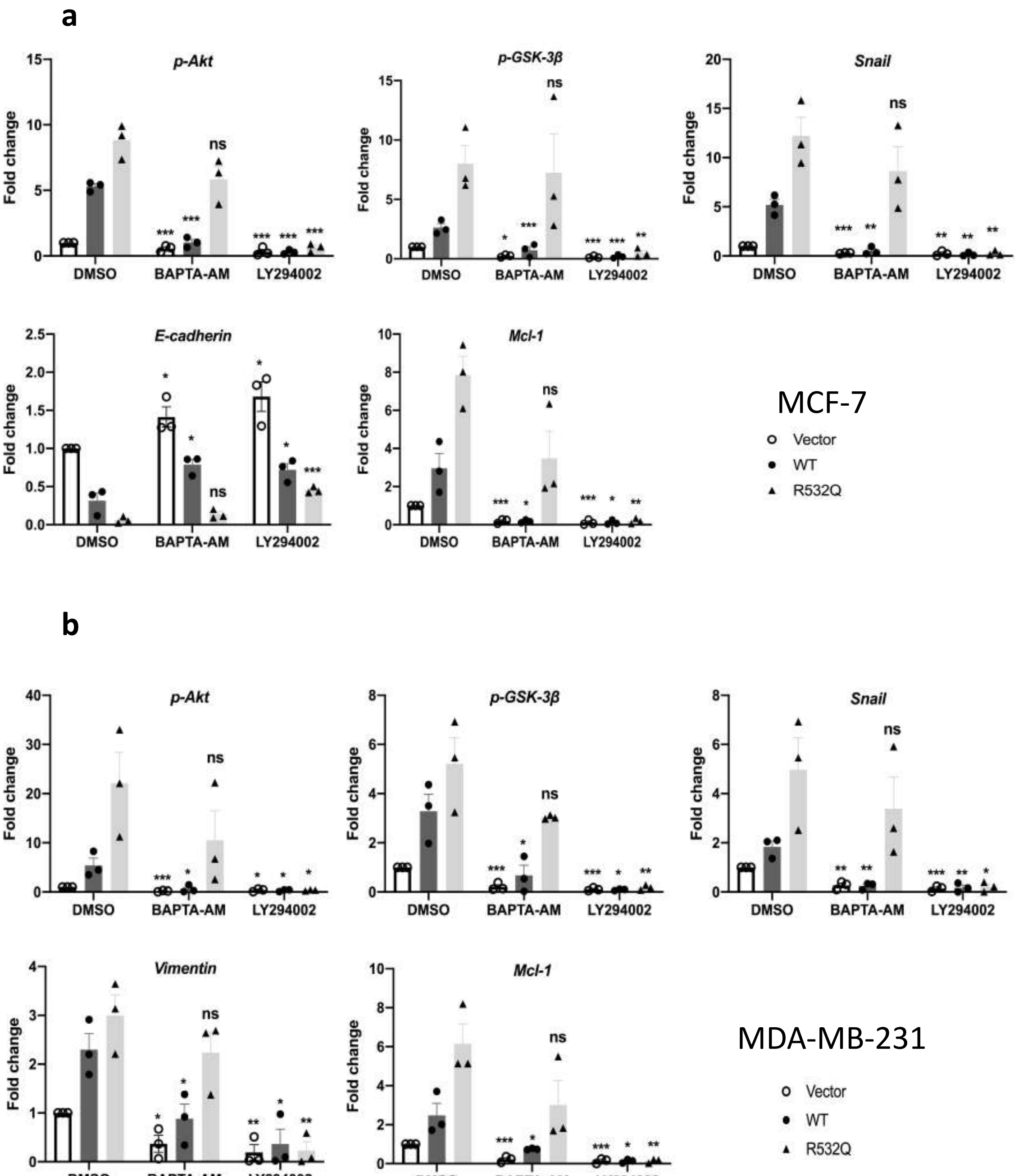

Supplementary Figure 9. Bar charts showing the effects of mutation R532Q, BAPTA-AM and LY294002 on the expression of indicated proteins using Western blotting in MCF-7 (a) and MDA-MB-231 (b) cells. Shown values were averaged from three independent experiments. *, $p < 0.1$; **, $p < 0.01$; ***, $p < 0.001$; ns, not significant.

Fig. S10

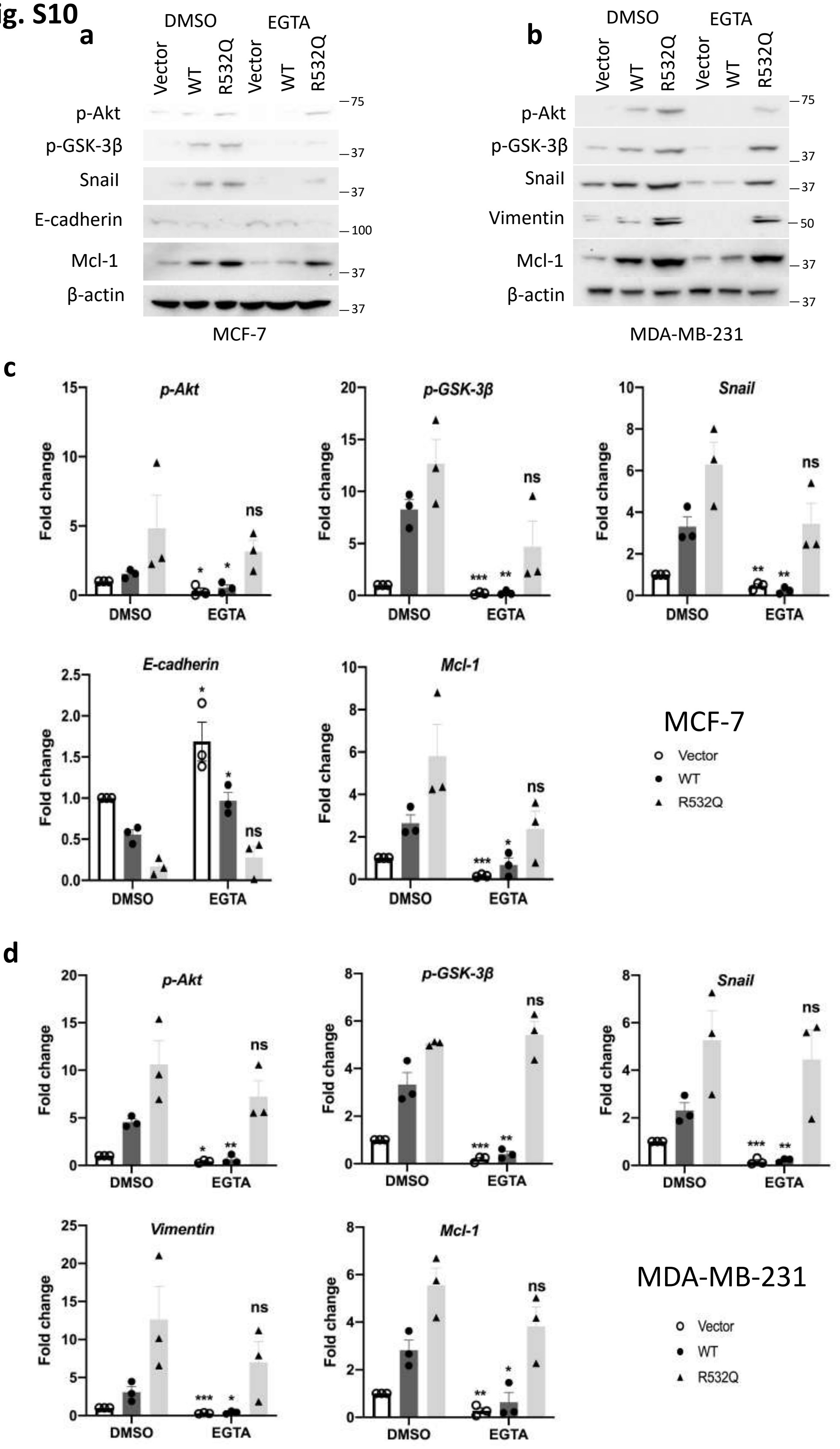
a
DMSO
EGTA
Vector
WT
R532Q
p-Akt
p-GSK-3β
Snail
E-cadherin
Mcl-1
β-actin
75
37
100
MCF-7
b
Vimentin
50
MDA-MB-231
c
Fold change
DMSO
EGTA
ns
E-cadherin
Mcl-1
MCF-7
Vector
WT
R532Q
d
MDA-MB-231

56

Supplementary Figure 10. Representative images showing the effects of mutation R532Q and EGTA incubation (2 mM, 1 hr) on the expression of the indicated proteins in MCF-7 (a) and MDA-MB-231 (b) cells. Bar charts showing the effects of EGTA incubation on the expression of the indicated proteins in MCF-7 (c) and MDA-MB-231 (d) cells. Shown values were averaged from three independent experiments. *, $p < 0.05$; **, $p < 0.01$; ***, $p < 0.001$.

Fig. S11

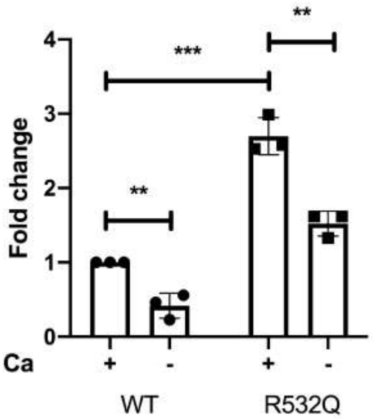

Supplementary Figure 11. Bar charts showing the effect of Ca on the amount of p85 precipitated by TRPV6 or mutant R532Q in MDA-MB-231 cells. Shown values were averaged from three independent experiments. **, $p < 0.01$; ***, $p < 0.001$.

Fig. S12

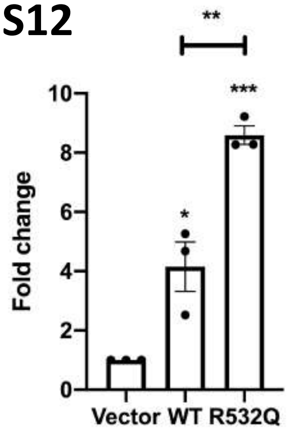

Supplementary Figure 12. Bar charts showing the effect of the R532Q mutation on

the membrane bound p85 inMDA-MB-231 stable cells. Shown values were averaged from three independent experiments.*, $p < 0.05$; **, $p < 0.01$; ***, $p < 0.001$.

Fig. S13

a

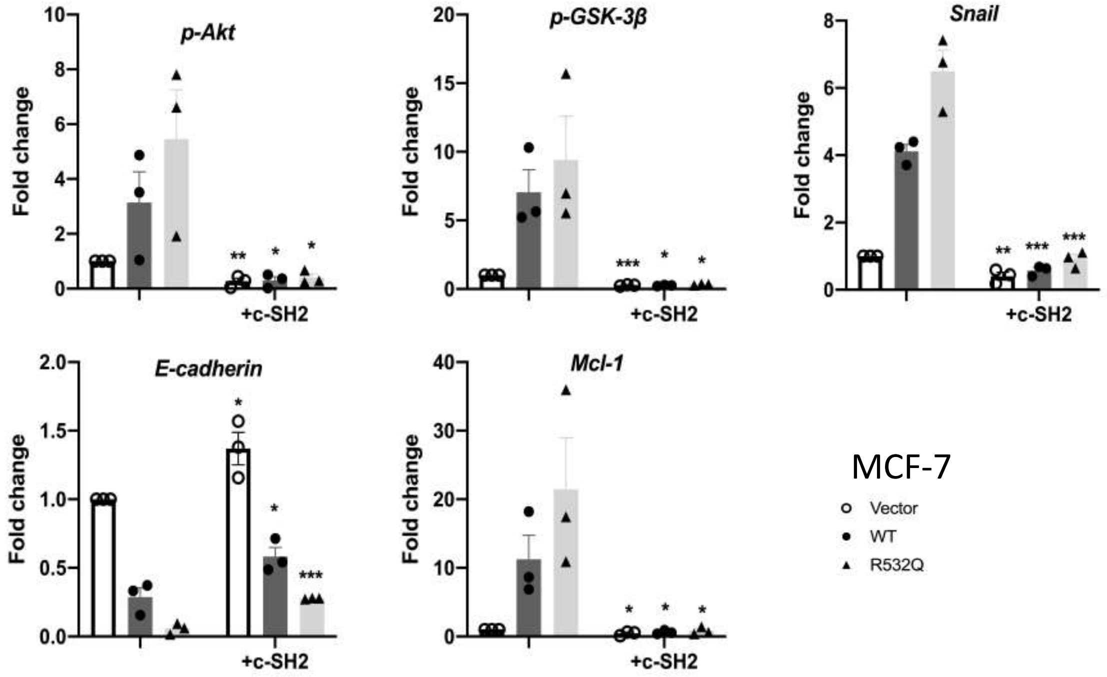
p-Akt
p-GSK-3β
Snail
E-cadherin
Mcl-1
Fold change
+c-SH2
MCF-7
Vector
WT
R532Q

b

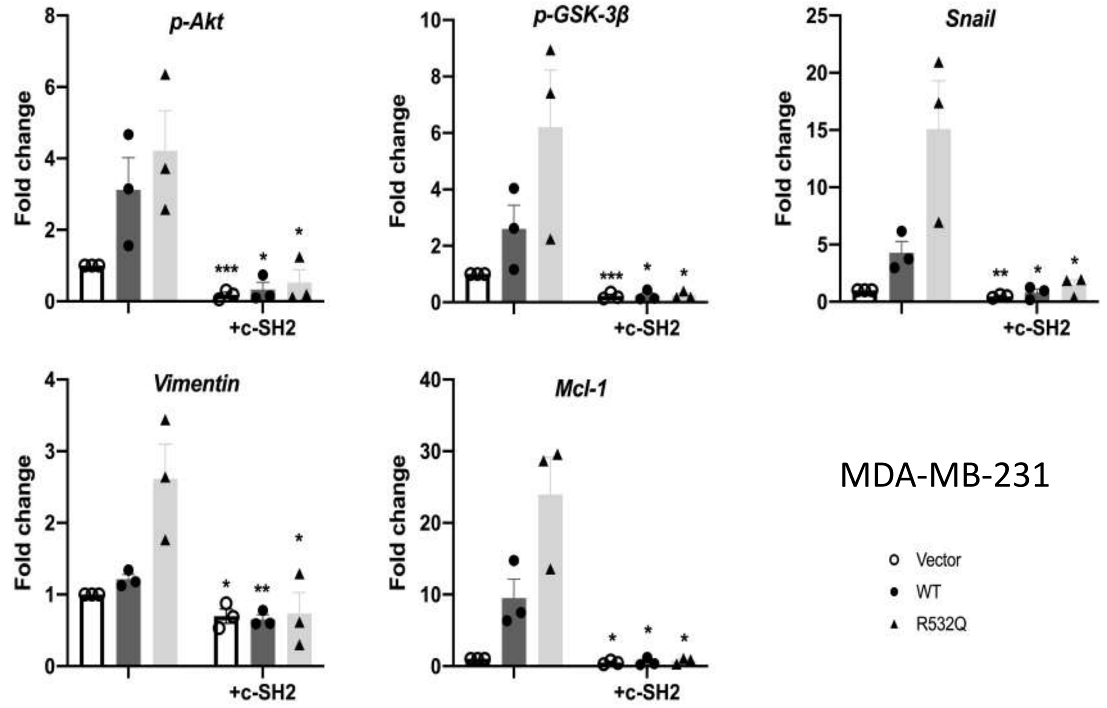
p-Akt
p-GSK-3β
Snail
Vimentin
Mcl-1
Fold change
+c-SH2
MDA-MB-231
Vector
WT
R532Q

Supplementary Figure 13. Bar charts showing the effects of mutation R532Q and c-SH2 on the expression of the indicated proteins in in MCF-7 (a) and MDA-MB-231 (b) cells by means of Western blotting. Shown values were averaged from three independent experiments. *, $p < 0.05$; **, $p < 0.01$; ***, $p < 0.001$.

Fig. S14

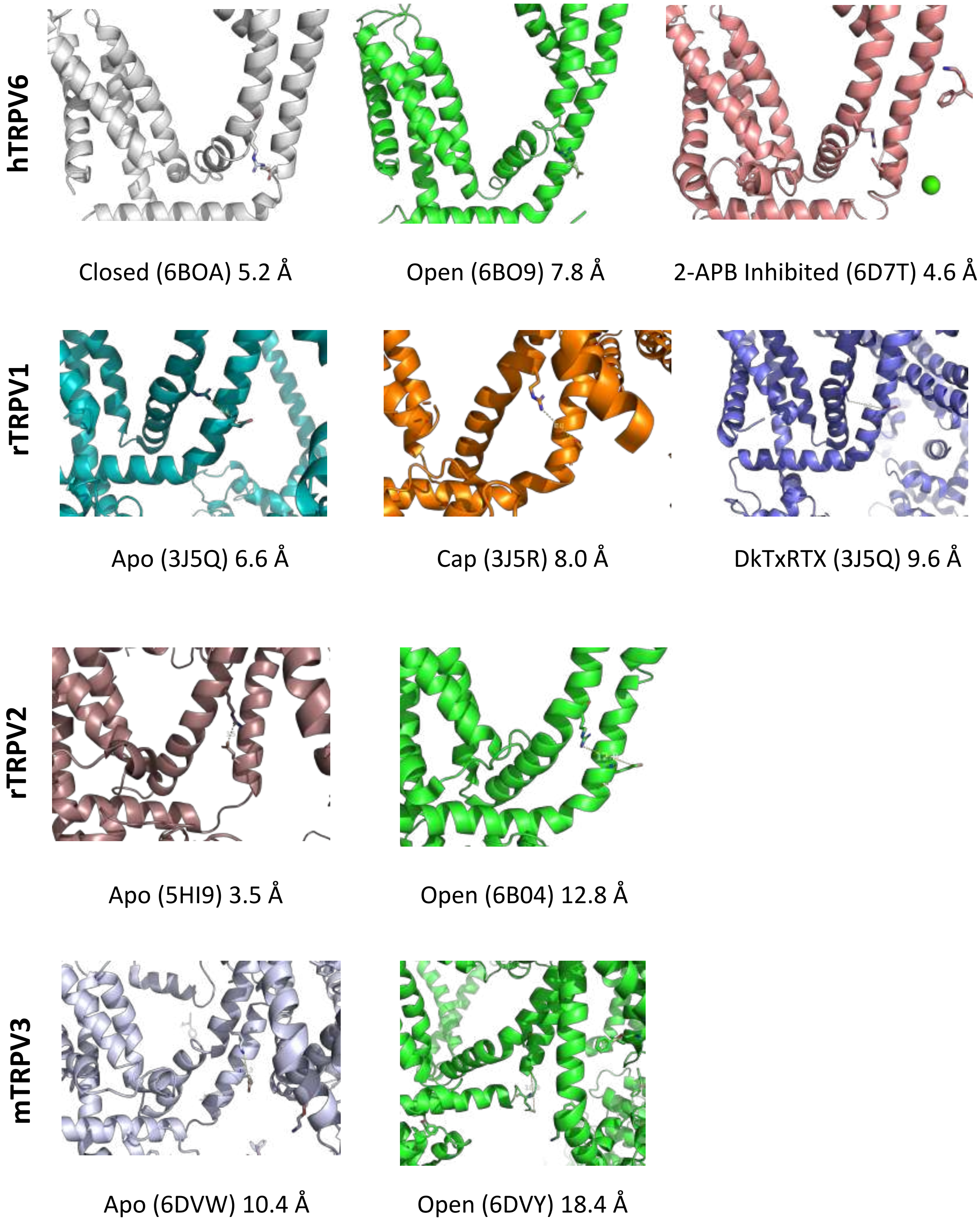

Supplementary Figure 14. Summary of conformational arrangements in TRP channels. Structural data of TRPV channels in the indicated states showing the distance between R532 and D620 in TRPV6 and the corresponding residues in other TRPV channels.

Supplementary Figure 15. Uncropped WB for main figures.

Fig. 1b

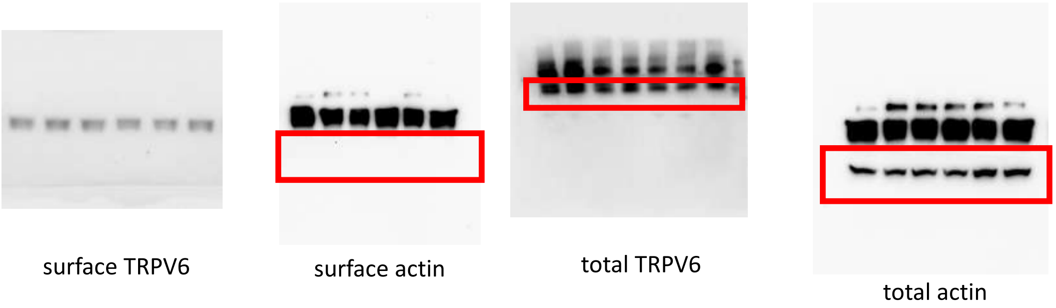

Fig. 1d

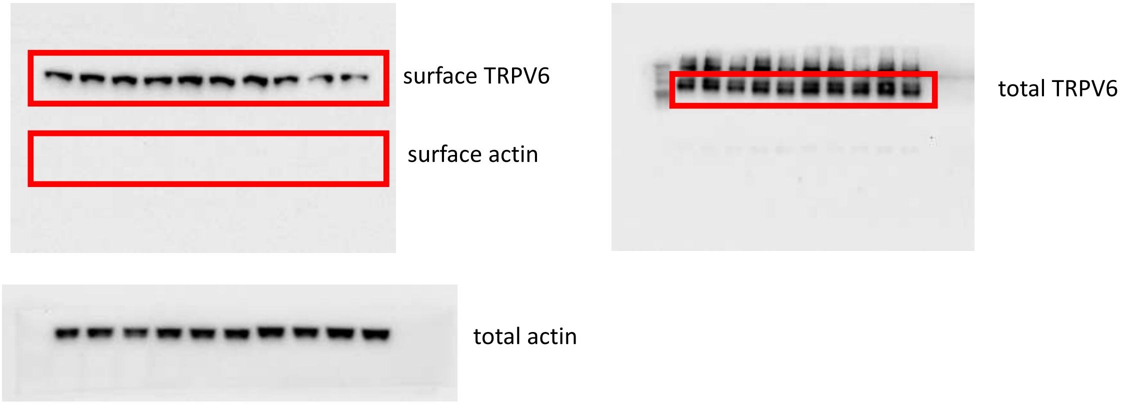

Fig. 2c

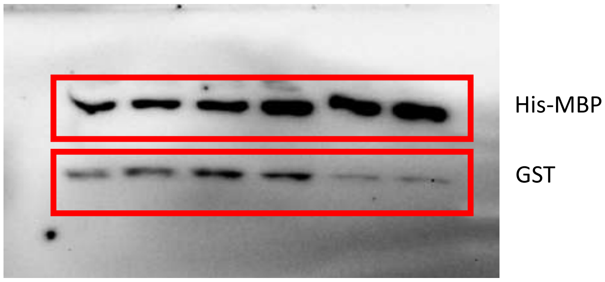

Fig. 4a

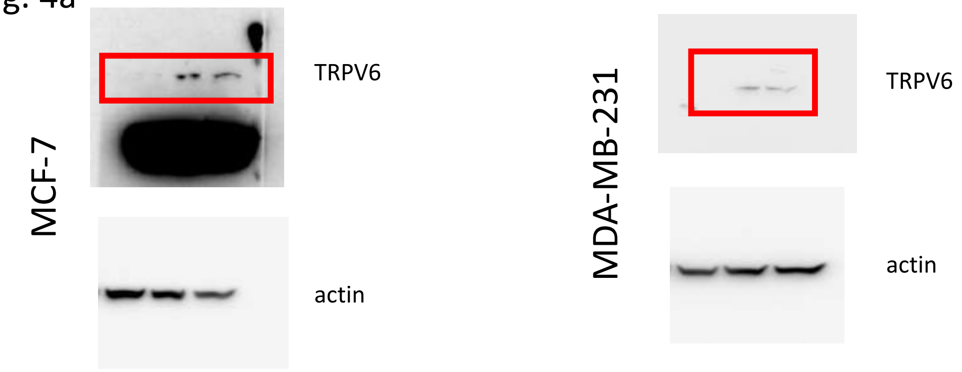

Fig. 5a

86

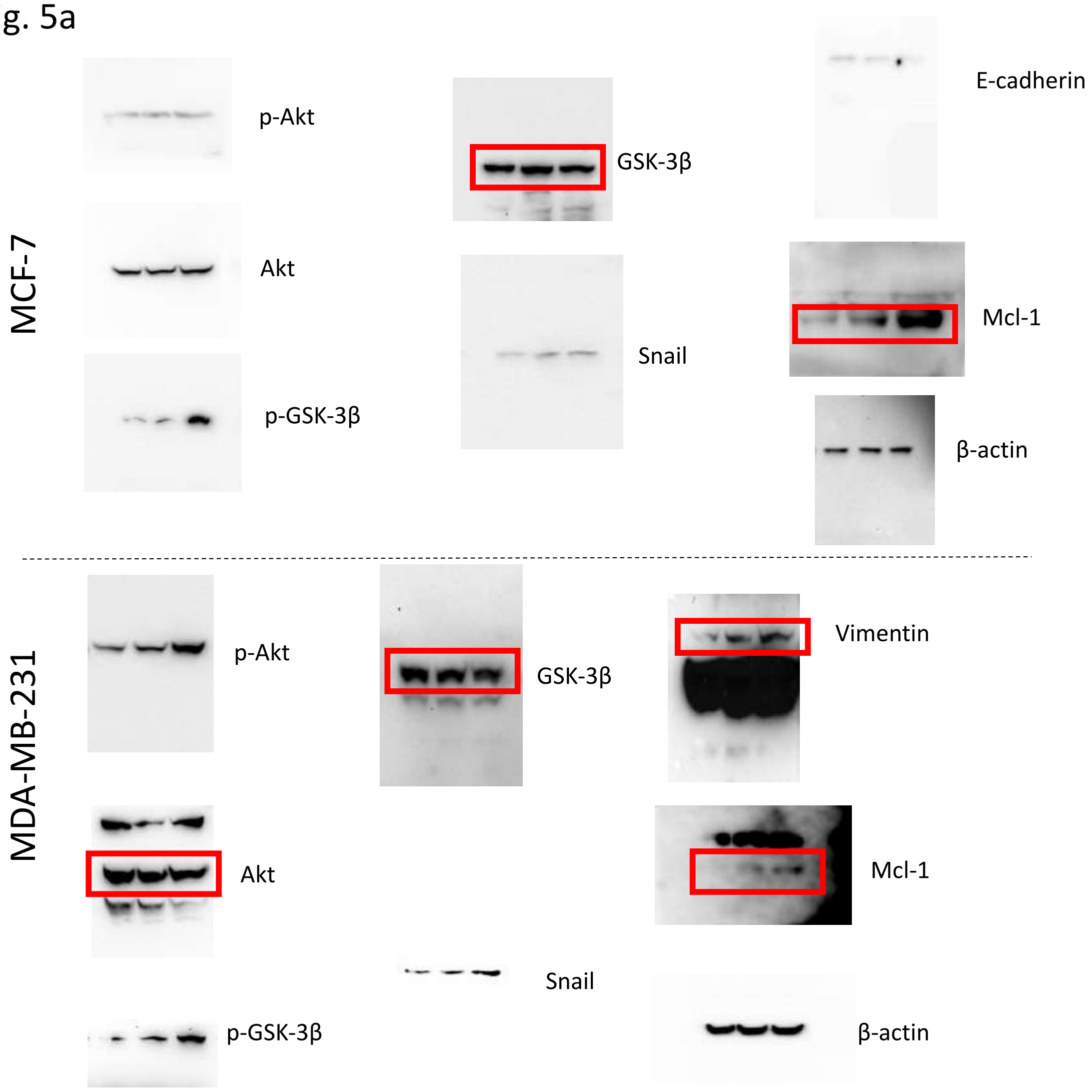

Fig. 5d

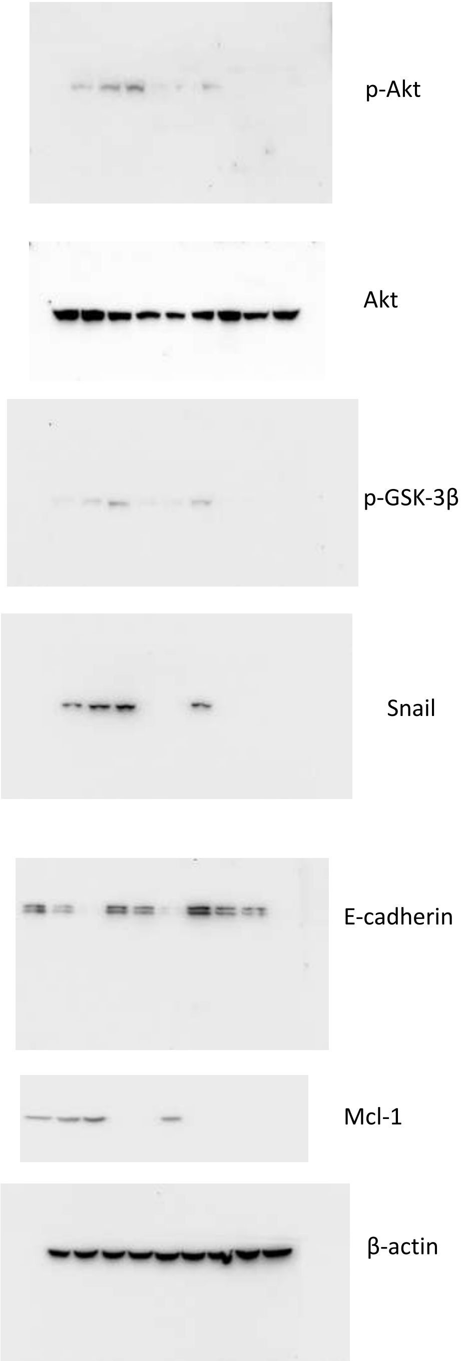

87

Fig. 5e

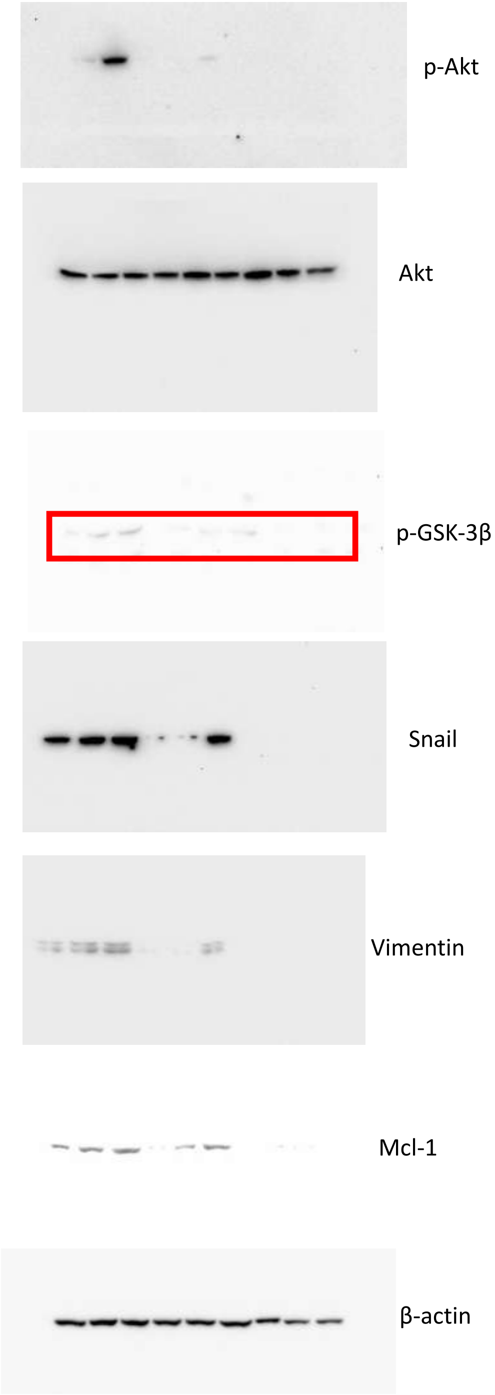

Fig. 6a

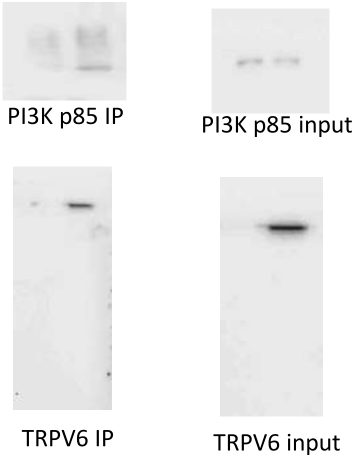

Fig. 6b

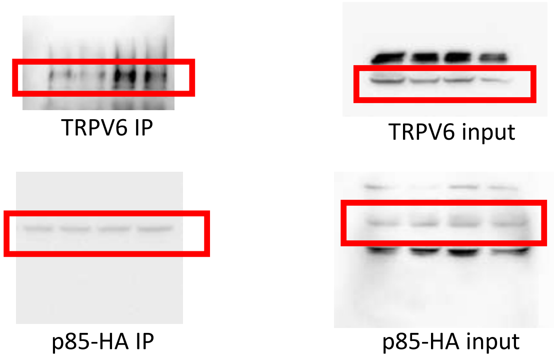

Fig. 6c

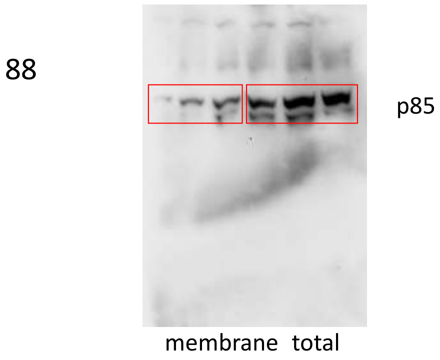

Fig. 6d

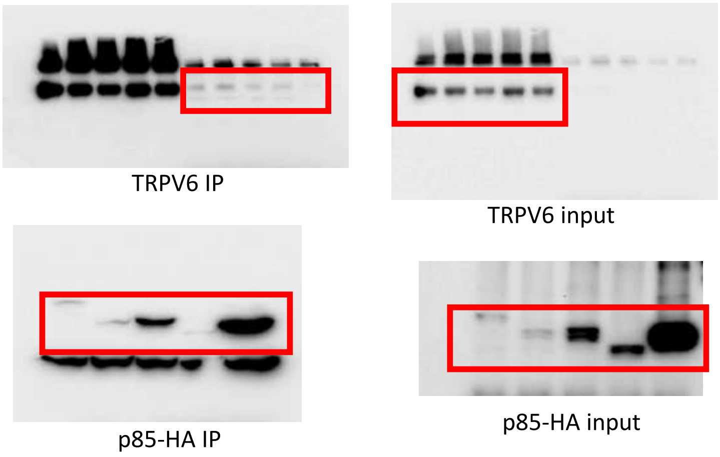

Fig. 6e

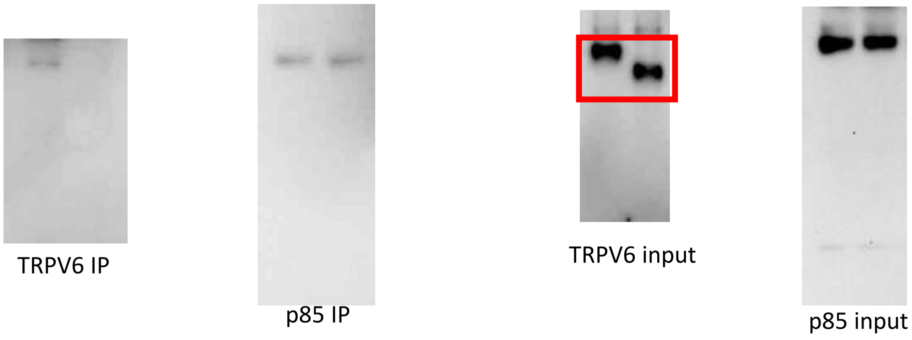

Fig. 6f

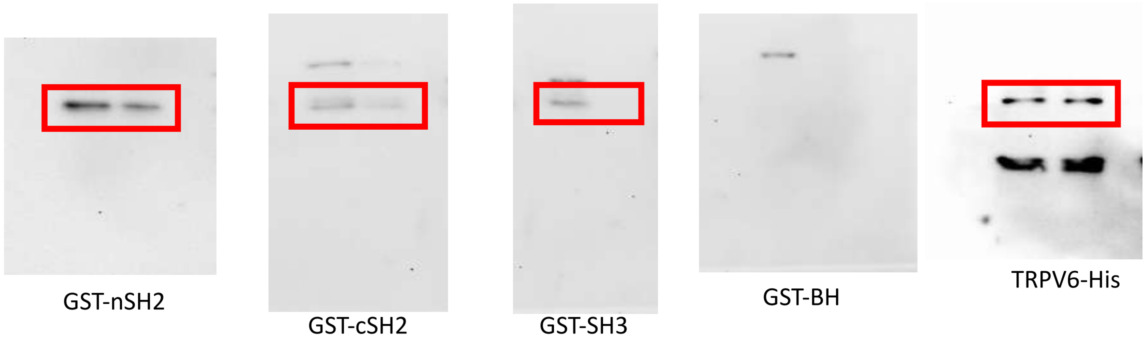

Fig. 6g

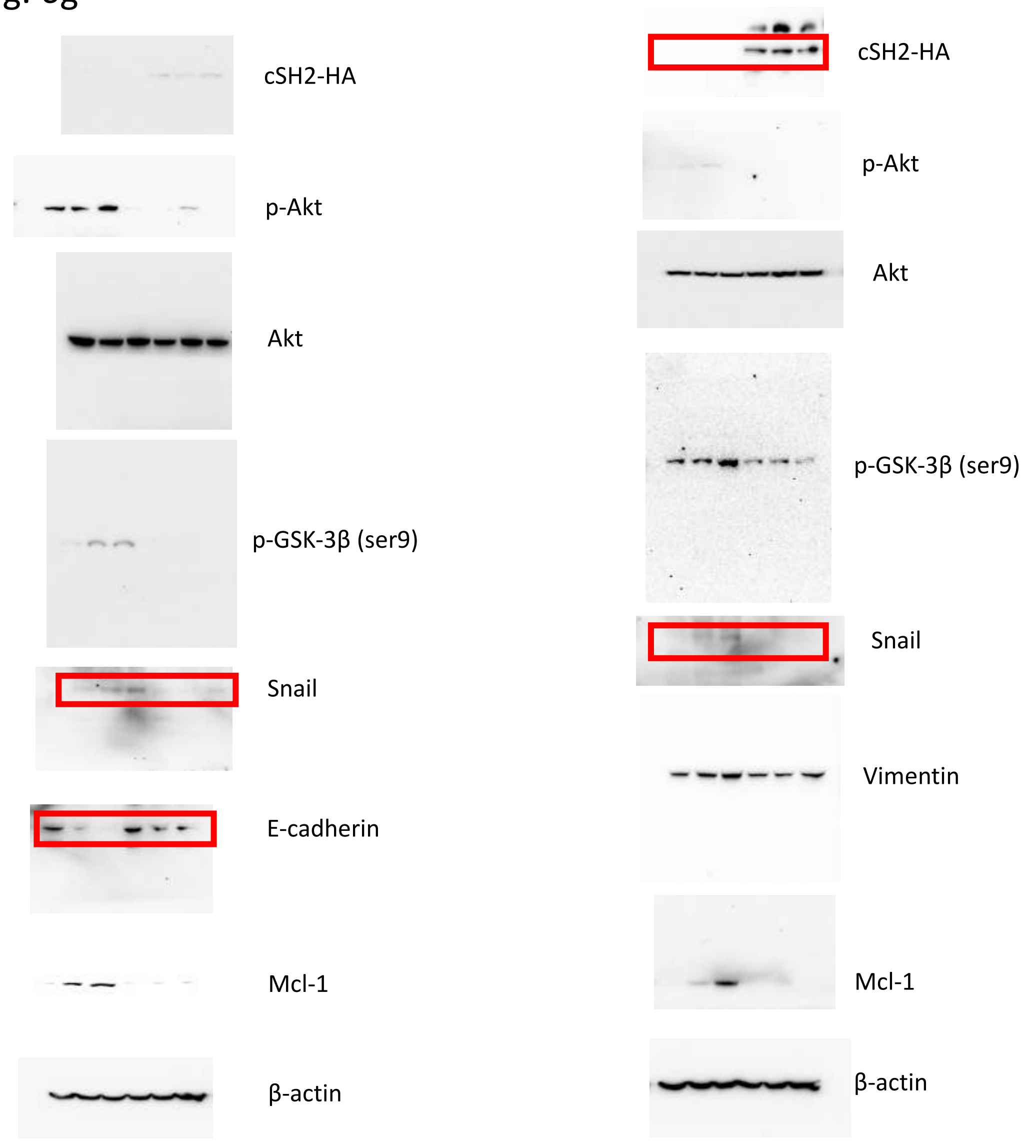

89
