# Supplementary material for: Auto-inhibitory intramolecular S5/S6 interaction in the TRPV6 channel regulates breast cancer cell migration and invasion
Source: Commun Biol. 2021 Aug 19;4:990. doi: 10.1038/s42003-021-02521-3 (PMC8376870; doi:10.1038/s42003-021-02521-3)
Supplement: Supplementary file 3 — Description of Supplementary Files [file 42003_2021_2521_MOESM3_ESM.pdf]

Description of Additional Supplementary Files

File Name: Supplementary Data 1
Description: Source data for graphs and charts.
