# Supplementary material for: Auto-inhibitory intramolecular S5/S6 interaction in the TRPV6 channel regulates breast cancer cell migration and invasion
Source: Commun Biol. 2021 Aug 19;4:990. doi: 10.1038/s42003-021-02521-3 (PMC8376870; doi:10.1038/s42003-021-02521-3)
Supplement: Supplementary file 5 — Reporting Summary [file 42003_2021_2521_MOESM5_ESM.pdf]

nature research

Corresponding author(s): Xing-Zhen Chen

Last updated by author(s): Jul 8, 2021

Reporting Summary

Nature Research wishes to improve the reproducibility of the work that we publish. This form provides structure for consistency and transparency in reporting. For further information on Nature Research policies, see our Editorial Policies and the Editorial Policy Checklist.

Statistics

For all statistical analyses, confirm that the following items are present in the figure legend, table legend, main text, or Methods section.

n/a	Confirmed	
☐	☒	The exact sample size (*n*) for each experimental group/condition, given as a discrete number and unit of measurement
☐	☒	A statement on whether measurements were taken from distinct samples or whether the same sample was measured repeatedly
☐	☒	The statistical test(s) used AND whether they are one- or two-sided<br>*Only common tests should be described solely by name; describe more complex techniques in the Methods section.*
☒	☐	A description of all covariates tested
☒	☐	A description of any assumptions or corrections, such as tests of normality and adjustment for multiple comparisons
☐	☒	A full description of the statistical parameters including central tendency (e.g. means) or other basic estimates (e.g. regression coefficient) AND variation (e.g. standard deviation) or associated estimates of uncertainty (e.g. confidence intervals)
☐	☒	For null hypothesis testing, the test statistic (e.g. *F*, *t*, *r*) with confidence intervals, effect sizes, degrees of freedom and *P* value noted<br>*Give P values as exact values whenever suitable.*
☒	☐	For Bayesian analysis, information on the choice of priors and Markov chain Monte Carlo settings
☒	☐	For hierarchical and complex designs, identification of the appropriate level for tests and full reporting of outcomes
☒	☐	Estimates of effect sizes (e.g. Cohen's *d*, Pearson's *r*), indicating how they were calculated

Our web collection on statistics for biologists contains articles on many of the points above.

Software and code

Policy information about availability of computer code

Data collection	Electrophysiology data collected by pClamp 9; calcium imaging data collected by MetaFluor. MD simulation analysis carried out with CPPTRAJ program of AMBER18.
Data analysis	Data were analyzed and plotted by GraphPad Prism 8.

For manuscripts utilizing custom algorithms or software that are central to the research but not yet described in published literature, software must be made available to editors and reviewers. We strongly encourage code deposition in a community repository (e.g. GitHub). See the Nature Research guidelines for submitting code & software for further information.

Data

Policy information about availability of data

All manuscripts must include a data availability statement. This statement should provide the following information, where applicable:

- Accession codes, unique identifiers, or web links for publicly available datasets
- A list of figures that have associated raw data
- A description of any restrictions on data availability

The data that support the findings of this study are available from the corresponding author (Dr. XZ Chen) upon request.

Field-specific reporting

Life sciences study design

All studies must disclose on these points even when the disclosure is negative.

Sample size	Sample size was determined to be sufficient for statistical analysis between groups. A biological triplicate or quadruplicate of experiments were typically performed prior to compilation of data for analysis. Oocyte and cultured cell numbers are described in figure legends where applicable.
Data exclusions	No data were excluded from statistical analyses.
Replication	Experiments were replicated using oocytes or cultured cells from at least 3 independent repeats. Exact numbers are given in figure legends.
Randomization	Xenopus oocytes and cultured cells were randomly grouped for experiments. All experiments were performed with appropriate negative controls, as well as positive controls where appropriate.
Blinding	Investigators were not blinded during data collection and analysis. Reported data were not subjective and were based on quantitative methods.

Reporting for specific materials, systems and methods

We require information from authors about some types of materials, experimental systems and methods used in many studies. Here, indicate whether each material, system or method listed is relevant to your study. If you are not sure if a list item applies to your research, read the appropriate section before selecting a response.

Materials & experimental systems

n/a	Involved in the study	
☐	☒	Antibodies
☐	☒	Eukaryotic cell lines
☒	☐	Palaeontology and archaeology
☒	☐	Animals and other organisms
☒	☐	Human research participants
☒	☐	Clinical data
☒	☐	Dual use research of concern

Methods

n/a	Involved in the study	
☒	☐	ChIP-seq
☒	☐	Flow cytometry
☒	☐	MRI-based neuroimaging

Antibodies

Antibodies used	Information for antibodies were described in Methods. Additional information is as follows: TRPV6 (custom acquired); β-actin (sc-47778, C4), Mcl-1 (sc-12756, 22), p85 (sc-374534, D-9) from Santa Cruz; p-Akt (4060S, D9E), Akt (9272, N/A), p-GSK-3β (5558S, D85E2), GSK-3β (12456T, D5C5Z), Snail (3895S, L70G2), e-cadherin (3195T, 24E10), vimentin (5741T, D21H3), HA-tag (3724S, C29F4) GST (2625T, 91G1) and His (2365S, N/A) from Cell Signaling Technology.
Validation	Each primary antibody is validated by the commercial supplier(s). TRPV6 antibody was previously validated by us (PMID: 12765696).

Eukaryotic cell lines

Policy information about cell lines

Cell line source(s)	MCF-7 and MDA-MB-231 cells are gifts from Zhixiang Wang lab, University of Alberta, Edmonton.
Authentication	None of the cell lines were authenticated.
Mycoplasma contamination	No mycoplasma contamination detected by immunoflurorescence DAPI staining with complete DAPI localization in the nucleus.
Commonly misidentified lines (See ICLAC register)	No cell line used is listed in the database of commonly misidentified cell lines.
